# Enterohemorrhagic *Escherichia coli* O157:H7 Infection Inhibits Host Endoplasmic Reticulum Stress in Intestinal Epithelial Cells via the PERK Pathway

**DOI:** 10.3390/pathogens14050440

**Published:** 2025-04-30

**Authors:** Litai Xu, Song Liang, Yaoguo Wang, Min Gao, Bao Zhang, Wei Zhao, Ying Hua, Chengsong Wan

**Affiliations:** 1 BSL-3 Laboratory (Guangdong), Guangdong Provincial Key Laboratory of Tropical Disease Research, School of Public Health, Southern Medical University, Guangzhou 510000, China; 3170090174@smu.edu.cn (L.X.); 18649381967@163.com (Y.W.); gaominms@163.com (M.G.); zhang20051005@126.com (B.Z.); zhaowei@smu.edu.cn (W.Z.); 2 Department of Laboratory Medicine, Shenzhen Children’s Hospital, Shenzhen 518000, China; lyric_439@smu.edu.cn; 3 Department of Laboratory Medicine, Nanfang Hospital, Southern Medical University, Guangzhou 510000, China

**Keywords:** Enterohemorrhagic *Escherichia coli*, EspF protein, endoplasmic reticulum stress, PERK pathway

## Abstract

Enterohemorrhagic *Escherichia coli* (EHEC) O157:H7 is a foodborne pathogen that causes a variety of diseases, ranging from self-limiting gastroenteritis to life-threatening extra-intestinal diseases such as hemolytic uremic syndrome. EspF, an effector protein secreted by the type III secretion system of EHEC, is primarily responsible for the development of inflammatory colitis. Our previous study revealed that EspF interacts with the host Annexin A6 (ANXA6) protein and targets the endoplasmic reticulum (ER). Given the critical effects of ER stress on the host responses of gastroenteritis, we explored the role of EspF–ANXA6 interaction in ER stress. Caco-2 cells were infected with different strains of EHEC and transfected with modified plasmids to establish in vitro research models. Our results revealed that infection with *espF*-deletion EHEC strains significantly exacerbated ER stress. Specifically, the phosphorylation of eIF2α was elevated, and the expression levels of BiP, ATF4, and CHOP were increased by more than 15% compared to those in cells infected with wild-type EHEC strains. Further experiments showed that EspF co-localizes with BiP and down-regulates the PERK pathway. Meanwhile, the EspF–ANXA6 interaction could aggravate the inhibition of the PERK pathway and stimulate calcium influx to disturb ER homeostasis, eventually leading to apoptosis. Our findings suggest that the EspF–ANXA6 interaction could inhibit ER stress through the PERK pathway, which may limit cell-to-cell communication and block the clearance of bacteria in host cells.

## 1. Introduction

Enterohemorrhagic *Escherichia coli* (*E. coli*) O157:H7 is a gastrointestinal pathogen that was first detected in the United States in 1982 [1]. Since then, sporadic food poisoning outbreaks throughout the world have been reported. According to reports from the US Centers for Disease Control and Prevention [2], there are up to 175,000 cases of Shiga toxin–producing *Escherichia coli* (STEC) infection every year, and approximately 36% of cases are caused by the strain O157:H7. More importantly, the number of cases is rising. The symptoms of most EHEC infections are mild, characterized by vomiting, diarrhea, and fever. It has considerable potential to cause life-threatening diseases in susceptible individuals, including hemolytic uremic syndrome and thrombotic thrombocytopenic purpura [3]. Recent studies have revealed that chronic gastroenteritis resulting from EHEC infection is associated with colon cancer [4], the second most lethal malignancy, and the third most commonly diagnosed type of cancer [5]. To minimize or eradicate adverse effects on public health, the pathogenesis of EHEC has been the focus of numerous epidemiological, microbiological, genomic, forensic, and diagnostic studies [6,7].

As extracellular bacteria, EHEC can secrete effector proteins through the type Ⅲ secretion system into host intestinal epithelial cells to form attachment and effacement (A/E) lesions [8], which promote the colonization of the intestinal epithelium by bacteria, disrupt the cytoskeleton, and finally cause disease [8,9,10]. EspF, a key effector protein encoded by the locus of the enterocyte effacement (LEE) pathogenicity island in *E. coli* O157:H7, executes pleiotropic virulence functions through host–protein interactions. These include the subversion of cellular metabolism, disruption of epithelial barrier integrity, inhibition of phagocytic clearance, induction of genomic instability, and provocation of mitochondrial dysfunction [11,12].

Recent studies revealed that the synthesis and processing of proteins by the endoplasmic reticulum (ER) influenced the secretory capacity of the intestinal epithelium [13,14]. The ER has important roles in protein folding, calcium storage, and lipid metabolism. When pathogenic bacteria invade cells and disrupt host homeostasis and immune defense, this results in ER stress. Cells then initiate the three arms of the unfolded protein response (UPR), which is a crucial pathway for maintaining homeostasis and normal ER function in eukaryotic cells [15]. The UPR comprises three main branches, including PKR-like ER kinase (PERK), inositol-requiring enzyme 1 (IRE1), and activating transcription factor 6 (ATF6), which regulate the production and/or quality of basic leucine zipper (bZIP) transcription factors to orchestrate the major regulatory circuits and ensure ER homeostasis [16,17]. Under conditions of prolonged stress due to bacterial or viral infection, attenuation of ER stress responses impairs the activation of UPR signaling cascades, which leads to activation of the apoptosis pathway and elimination of the damaged cells [18]. Indeed, a lack of normal UPR activity contributes to gut inflammation and IBD susceptibility [19,20]. Accumulating evidence indicates that PERK, a potential therapeutic target for treating intestinal inflammation caused by STEC infection, activates autophagy via its downstream transcription factor ATF4 to degrade invading pathogens and suppress inflammation [21,22]. EspF could target host organelles such as the ER and the ER membrane to alter the normal functioning of various pathways in EHEC-infected host cells [23]. However, the pathogenic mechanism by which EspF causes ER stress remains unclear. In addition to EspF working alone, the capacity of EspF to collaborate with host proteins plays an important role in the pathogenic mechanism of EHEC O157:H7. An accumulating number of papers have reported that interactions of virulence effectors with host receptors have influenced protein-folding diseases [20,24,25]. Our previous study revealed that EspF and the host protein ANXA6 were strongly connected [23]. ANXA6 is a candidate physiological modulator of the Ca^2+^ release channel and the scaffolding of specific proteins or multifarious protein complexes, closely related to the ER stress [26]. In addition, it was reported that ANXA2 exhibited positive effects on the IRE1-XBP1 pathway during *P. aeruginosa* infection [27]. Therefore, we speculate that there is a mutual effect between EspF and ANXA6, a host protein that regulates ER stress to control the development of infectious diseases.

To confirm these conjectures, we performed in vitro experiments to verify the relationship between EspF and ER stress. By infecting Caco-2 cells with either a wild-type EHEC strain or its Δ*espF* mutant and expressing EspF or EspF–ANXA6 fusion proteins, we explored the role of the EspF–ANXA6 interaction in EHEC O157:H7-induced ER stress. We found that the EspF–ANXA6 interaction disrupts ER homeostasis through the PERK pathway. An improved understanding of the relationship between ER stress and EspF could aid in the development of novel therapeutic strategies.

## 2. Materials and Methods

### 2.1. Cells Culture and Plasmids

Caco-2 cells and HeLa cells were kindly gifted by Professor Bao Zhang. Cells were incubated in Dulbecco’s modified Eagle’s medium (DMEM) (Thermo Fisher Scientific, Waltham, MA, USA) supplemented with 10% fetal bovine serum (FBS) (Thermo Fisher Scientific, Waltham, MA, USA) and 100 U/I penicillin and streptomycin (Thermo Fisher Scientific, Waltham, MA, USA), at 37 °C under 5% CO_2_. For transient transfection, the following plasmids were generated in our laboratory: pEGFP (E), pEGFP-EspF (EE), and pEYFP-EspF-T2A-ANXA6 (EYEA). Caco-2 cells and HeLa cells were transfected using Lipofectamine 3000 (Thermo Fisher Scientific, Waltham, MA, USA).

### 2.2. Bacterial Infection

For infection, the following strains were preserved in our laboratory: wild-type EHEC O157:H7 EDL933 and *espF*-deletion strains (Δ*espF*). Bacterial strains were grown at 37 °C in Luria–Bertani broth overnight. The Δ*espF* strains were cultured with an appropriate concentration of kanamycin (Sigma-Aldrich, St. Louis, MO, USA). Overnight bacterial cultures were diluted in serum (2%) and an antibiotic-free mixture of DMEM and grown to the mid-log growth phase. Monolayers were infected with *E. coli* at an MOI of 100 for indicated times.

### 2.3. Western Blot

For protein isolation, cells were washed with PBS (Solarbio, Beijing, China) three times on the ice and lysed in radioimmunoprecipitation assay lysis buffer (Beyotime, Shanghai, China) containing PMSF (Beyotime, Shanghai, China) and phosphorylase inhibitor cocktail (Bimake, Houston, TX, USA). The protein concentration was measured using the bicinchoninic acid assay. Proteins were boiled in SDS sample buffer and separated by SDS-PAGE. The proteins were transferred to PVDF membranes (Millipore, Burlington, MA, USA), which were blocked with 5% skimmed milk in Tris-buffered saline with 0.1% Tween 20. Membranes were probed at 4 °C overnight with the following primary antibodies (1:1000): anti-BiP, anti-eIF2α, anti-p-eIF2α, anti-ATF4, anti-CHOP (Cell Signaling Technology, Danvers, MA, USA), and anti-β-actin (Proteintech, Wuhan, China). After that, the membranes were incubated with a goat anti-rabbit IgG secondary antibody or a goat anti-mouse IgG secondary antibody (Proteintech, Wuhan, China) for 1 h. Finally, the protein bands were visualized using an ECL chemiluminescence substrate kit (Millipore, MA, USA). Grayscale analysis was performed using ImageJ software (Version 1.54) (National Institutes of Health, Bethesda, MD, USA).

### 2.4. Immunofluorescence Assay

Caco-2 cells were plated on confocal dishes. When the cells reached 70% confluence, the cells were transfected with pEGFP (E), pEGFP-EspF (EE), or pEYFP-EspF-T2A-ANXA6 (EYEA). After transfection, the cells were washed three times and fixed in 4% paraformaldehyde for 15 min, followed by ventilation in 0.5% Triton-100 for 15 min. The fixed cells were blocked by goat serum for 30 min and stained with a primary antibody overnight at 4 °C, followed by incubation with a secondary antibody for 1 h. Nuclei were stained with DAPI for 5 min in the dark. Co-localization was monitored with a Zeiss LSM880 confocal microscope (Zeiss, Oberkochen, Germany) and coupled with ZEISS Zen software (Version 3.11) (Zeiss, Oberkochen, Germany) using a 63× oil immersion objective. Excitation and emission wavelengths were adjusted to avoid the carryover of fluorescence to other channels.

### 2.5. Calcium Imaging

After transfection, Caco-2 cells were gently washed with HBSS (Bioss, Woburn, MA, USA) three times and stained with 1 μM RHOD-2/AM (Yeasen, Shanghai, China) containing an appropriate concentration of Pluronic (Yeasen, Shanghai, China) diluted in HBSS for 15 min at 37 °C. To fully remove residual working reagents, the cells were washed with gentle shaking three times, and then they were covered with buffer for 20 min at 37 °C to ensure the AM body was completely esterified. Calcium imaging was performed with a Zeiss LSM880 confocal microscope (Zeiss, Oberkochen, Germany) at 40× magnification and coupled with ZEISS Zen software (Version 3.11) (Zeiss, Oberkochen, Germany). Excitation and emission wavelengths were adjusted to avoid the carryover of fluorescence to other channels.

### 2.6. Apoptosis Assay

After transfection, Caco-2 cells were gently collected and processed for Annexin V staining with a 633 Apoptosis Detection kit (DOJINDO, Kumamoto, Japan). The cells were digested by a pancreatic enzyme without EDTA and then washed with cold PBS two times. The final concentration of cell suspension was 1 × 10^6^ cells/mL in a Annexin V binding solution. The cells (in 100 μL) were stained with 5 μL Annexin V-633 and 5 μL of PE in Annexin V binding solution. After 15 min, 400 μL of Annexin V binding solution was added, and the cells were analyzed with a multi-dimensional HD flow cytometry analyzer (BD Biosciences, Franklin Lakes, NJ, USA) in 1 h.

### 2.7. Statistical Analysis

All statistical analyses were performed using SPSS (Version 26.0) (SPSS Inc., Chicago, IL, USA). The data were presented as mean ± SD and analyzed with the one-way ANOVA or two-way ANOVA method, followed by Dunnett’s test for separate comparisons. *p* < 0.05 was considered statistically significant.

## 3. Results

### 3.1. espF-Deletion O157:H7 Strain Infection Enhances ER Stress in Caco-2 Cells

**Figure 1:**
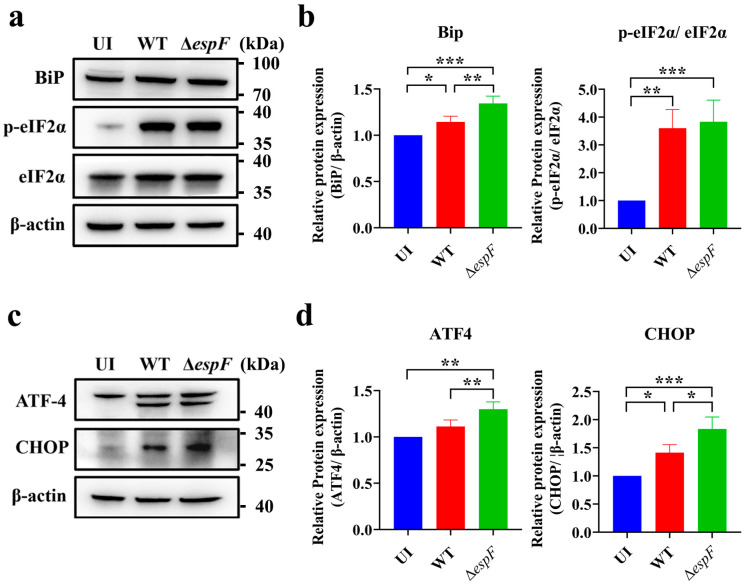
Infection with the *espF*-deletion O157:H7 strain increases ER stress in Caco-2 cells. (**a**) The expression levels of BiP, eIF2α, and p-eIF2α in Caco-2 cells infected with EHEC or Δ*espF* for 6 h were analyzed by Western blot. (**b**) Band intensities of BiP, eIF2α, and p-eIF2α were measured densitometrically. (**c**) Expression of proteins involved in the PERK pathway (ATF4 and CHOP) in infected Caco-2 cells. (**d**) Band intensities of ATF4 and CHOP were measured densitometrically. UI, uninfected Caco-2 cells; WT, Caco-2 cells infected with EHEC O157:H7 strains EDL933 and Δ*espF*, Caco-2 cells infected with *espF*-deletion O157:H7 strain. Full-length imprints/gels are shown in Supplementary Figures S1–S4. Data are shown as the mean ± SD from at least three independent experiments. Statistical analyses were performed using one-way ANOVA. * *p* < 0.05; ** *p* < 0.01 and *** *p* < 0.001.

To investigate the impact of EHEC O157:H7 on ER stress, we initially examined protein level alterations in Caco-2 cells infected with wild-type strains or the Δ*espF* mutant by using Western blot. The construction method of the in vitro bacterial strain infection model referred to previous studies [28], with a multiplicity of infection (MOI) of 100 and an infection duration of 6 h. BiP functions as an ER stress sensor and is considered an indicator of UPR activation [29]. Compared with the uninfected group, BiP levels were significantly increased in the wild-type group. Moreover, BiP levels were higher in the Δ*espF* group than in the wild-type group, with an increase of 17.35% (Figure 1a,b). This finding suggests that while EHEC O157:H7 infection induces ER stress, its effector protein EspF exerts an inhibitory effect on this cellular response.

To alleviate proteotoxic stress induced by misfolded proteins in the ER, eukaryotic cells activate the UPR. Given the crucial role of the PERK pathway in mitigating ER stress induced by STEC infection [21], we examined the expression levels of ATF4 and CHOP, as well as the phosphorylation status of eIF2α within the PERK pathway. As anticipated, our findings revealed that compared to the control group, infection with EHEC O157:H7 significantly increased the expression levels of ATF4 by 16.70% and CHOP by 29.84%. Moreover, the phosphorylation status of eIF2α was also markedly elevated (Figure 1a–d). These results indicate that in response to EHEC O157:H7 infection, the host activates the PERK pathway to alleviate ER stress induced by the pathogen. The same trend was observed in the Δ*espF* group. Additionally, the protein levels of ATF4 and CHOP were drastically increased in the Δ*espF* group compared with the wild-type group, suggesting that infection with the *espF*-deletion O157:H7 strain more strongly stimulates ER stress in Caco-2 cells. Collectively, these results indicate that EspF inhibits host ER stress and may modulate the PERK pathway.

### 3.2. EspF Protein Co-Localizes with BiP in Caco-2 Cells

Considering that infection with the *espF*-deletion O157:H7 strain could increase the BiP levels in Caco-2 cells, we hypothesized that EspF is associated with ER stress and down-regulates BiP. Our previous studies have demonstrated that the EHEC effector protein EspF interacts with host ANXA6 [23], a calcium-dependent membrane-binding protein that plays a crucial role in maintaining cellular homeostasis [30]. Given that the ER is the most important calcium reservoir in the cell [31] and that inositol 1,4,5-trisphosphate receptors (IP3Rs) on the ER membrane mediate the release of ER calcium, thereby participating in the dissociation and re-association equilibrium of the BiP-substrate complex [32], we generated mammalian expression plasmids encoding full-length EspF (EE) or an EspF–ANXA6 fusion protein (EYEA) to explore the effects of EspF-ANXA6 on ER stress.

**Figure 2:**
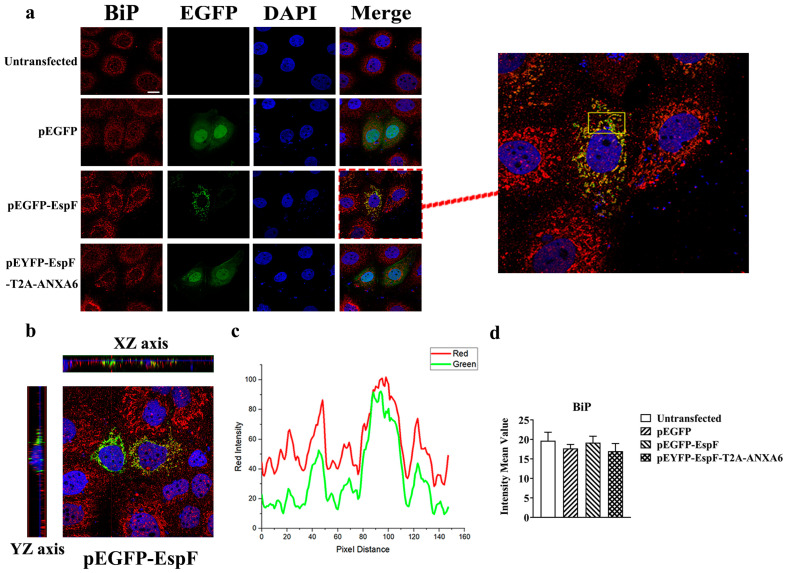
EspF co-localizes with BiP in Caco-2 cells. (**a**) Immunofluorescence microscopy of BiP protein in Caco-2 cells, which were transfected with the indicated plasmids for 48 h. Green indicates fluorescence expressed by the fluorescent plasmids pEGFP or pEYFP. The nucleus was labeled with DAPI (blue). BiP was labeled with anti-BiP (red). Images were taken using an LSM880 confocal laser scanning microscope at 630× magnification (scale bar: 20 μm). (**b**) The 3D distribution of BiP protein in Caco-2 cells in EE group. (**c**) Pixel distance between red and green fluorescence intensities. (**d**) Fluorescence intensity analysis of Bip protein in Caco-2 cells. Data are shown as the mean ± SD from at least three independent experiments. Statistical analyses were performed using one-way ANOVA.

Caco-2 cells were transfected to analyze the distribution and expression level of BiP using immunofluorescence. pEGFP (E) was used as a negative control. In EspF-expressing Caco-2 cells, EspF and BiP mainly overlapped in the cytoplasm, showing a scattered punctuate distribution (Figure 2a–c), which suggests that EspF co-localizes with BiP, the signature protein of ER stress. Nevertheless, compared with the EspF-expressing group, the degree of co-localization between EspF and BiP was less in EspF–ANXA6-expressing Caco-2 cells (Figure 2a–c). In addition, compared with the EYEA group, the EE group exhibited an absence of green fluorescence in the nuclear region (Figure 2a). This observation may indicate that ANXA6 alters the subcellular localization of EspF, thereby promoting its nuclear translocation. Contrary to expectations, we detected no significant difference in the content of BiP among the groups (Figure 2d). The above results verify that EspF co-localizes with BiP, which may play an important role in ER stress and ER homeostasis. However, the mechanism by which EspF-ANXA6 induces the subcellular relocalization of the EHEC effector protein EspF remains to be elucidated.

### 3.3. EspF–ANXA6 Interaction Inhibits ER Stress Through the PERK Pathway

**Figure 3:**
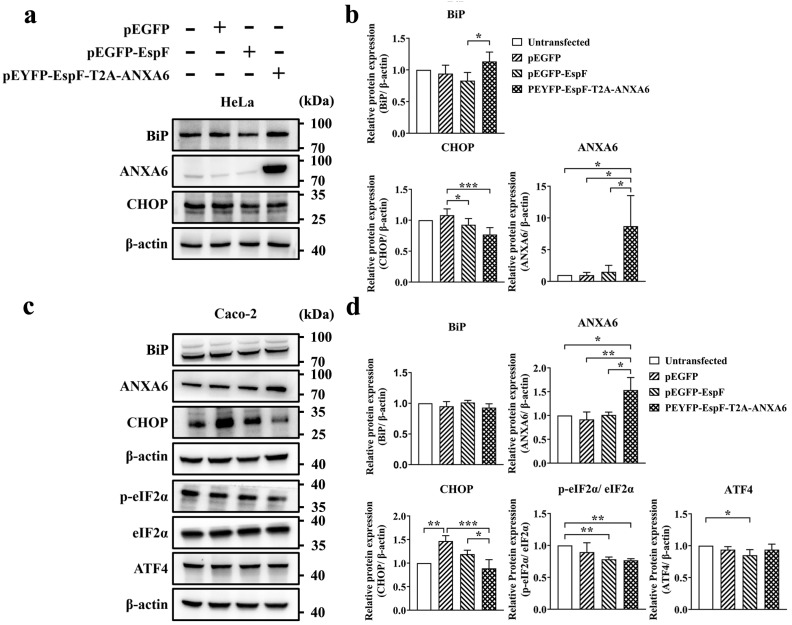
EspF–ANXA6 interaction inhibits ER stress through the PERK pathway. (**a**) The expression levels of BiP, ANXA6, and CHOP in HeLa cells transfected with the indicated plasmids for 48 h was analyzed by Western blot. (**b**) Band intensities of BiP, ANXA6, and CHOP were measured densitometrically. (**c**) The expression levels of BiP, ANXA6, CHOP, eIF2α, p-eIF2α, and ATF4 in Caco-2 cells transfected with indicated plasmids for 48 h were analyzed by Western blot. (**d**) Band intensities of BiP, ANXA6, CHOP, eIF2α, p-eIF2α, and ATF4 were measured densitometrically. Full-length imprints/gels are shown in Supplementary Figures S5–S12. Data are shown as the mean ± SD from at least three independent experiments. Statistical analyses were performed using one-way ANOVA. * *p* < 0.05; ** *p* < 0.01 and *** *p* < 0.001.

Based on the above observations, we hypothesized that the effector protein EspF could individually ameliorate ER stress. To shed light on the mechanisms underlying the effects of EspF on ER stress, we transfected Caco-2 cells and HeLa cells with an EspF expression plasmid (EE) or an EspF–ANXA6 fusion plasmid (EYEA). As depicted in Figure 3a,b, EspF exhibited a slight suppressive effect on BiP expression in HeLa cells (*p* > 0.05). Conversely, the BiP levels were slightly up-regulated when cells were transfected with the EspF–ANXA6 fusion plasmid (*p* > 0.05). Although these differences were not statistically significant, a discernible trend of altered expression levels was observed between the groups. This suggests that EspF may down-regulate the ER stress level, while the interaction between EspF and ANXA6 may exert the opposite effect. Furthermore, EspF down-regulated CHOP. CHOP is considered to be a downstream protein of the PERK pathway, which can induce ER stress-related apoptosis. This finding confirms that EspF inhibits ER stress and ER stress-related apoptosis in HeLa cells.

In contrast, Caco-2 cells transfected with either the EspF expression plasmid (EE) or EspF–ANXA6 fusion plasmid (EYEA) exhibited no significant alterations in BiP levels (Figure 3c,d), as corroborated by the immunofluorescence analysis. Nevertheless, Caco-2 cells treated with the EspF expression plasmid showed decreased CHOP levels. Moreover, the expression level of CHOP in cells transfected with EspF–ANXA6 fusion plasmid was significantly decreased compared to those treated with EspF alone. These findings show that EspF has little effect on BiP levels but inhibits ER stress-related apoptosis, which may depend on EspF–ANXA6 interaction.

The grayscale analysis additionally revealed an acute decrease in the eIF2α phosphorylation level in Caco-2 cells treated with the EspF expression plasmid or EspF–ANXA6 fusion plasmid, and a slight change in the ATF4 levels was observed in EspF-expressing Caco-2 cells (Figure 3c,d). The changes are consistent with the results from the bacterial infection experiments above. Based on these results, we conclude that EspF substantially inhibits ER stress and delays ER stress-related apoptosis in host cells via the PERK pathway in Caco-2 cells.

### 3.4. EspF–ANXA6 Interaction Stimulates Calcium Release and Promotes Cell Apoptosis

**Figure 4:**
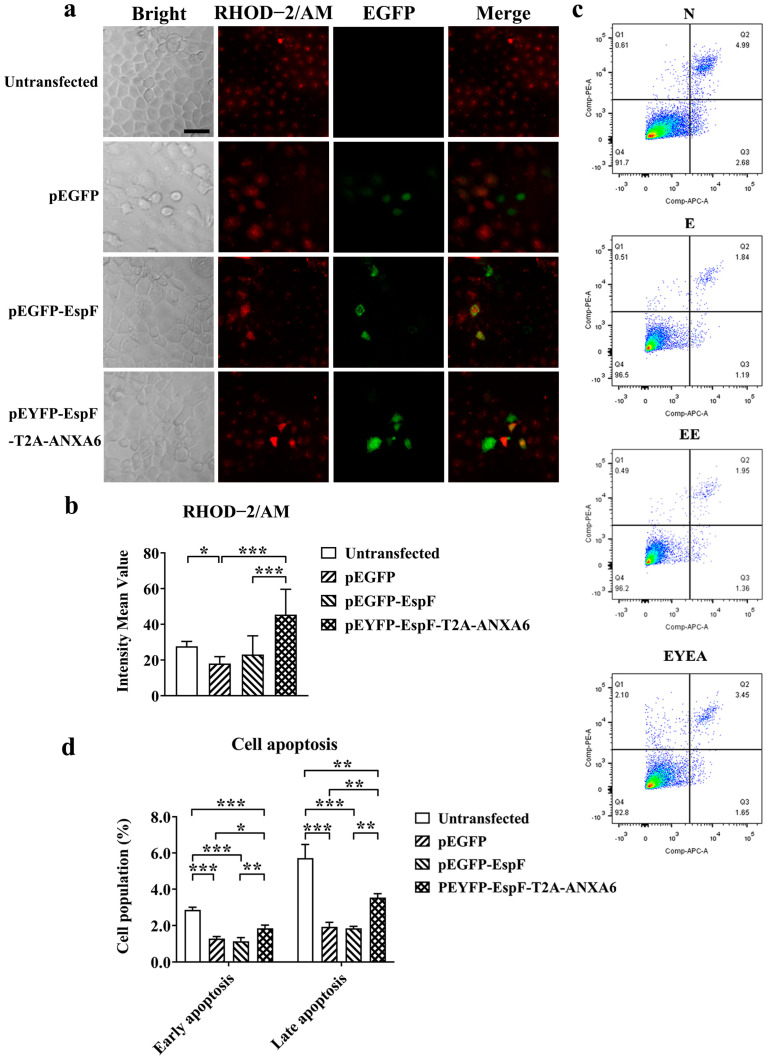
EspF–ANXA6 stimulates calcium release and induces apoptosis in Caco-2 cells. (**a**) Images of intracellular free calcium stained with RHOD-2/AM in Caco-2 cells, which were transfected with indicated plasmids for 48 h. Images were acquired by an LSM880 confocal laser scanning microscope at 400× magnification (scale bar: 50 μm). (**b**) Grayscale analysis of the fluorescence intensity of calcium in the cytoplasm. Statistical analyses were performed using one-way ANOVA. (**c**) Apoptosis was analyzed with an Annexin V-633/PE apoptosis detection kit, followed by flow cytometry. Q1, Q2, Q3, and Q4 represent PE-positive, Annexin V-/PE-positive, Annexin V-/PE-negative, and Annexin V-positive cells, respectively. (**d**) The proportions of early apoptotic cells (Q3) and late apoptotic cells (Q2) were analyzed statistically. Statistical analyses were performed using two-way ANOVA. N, untransfected cells; E, cells transfected with pEGFP; EE, cells transfected with pEGFP-EspF; and EYEA, cells transfected with pEYFP-EspF-T2A-ANXA6. Data are shown as the mean ± SD from at least three independent experiments. * *p* < 0.05; ** *p* < 0.01 and *** *p* < 0.001.

Given the pivotal role of ANXA6 in calcium homeostasis and membrane binding [33], we hypothesized that the interaction of EspF and ANXA6 may inhibit ER stress and disrupt ER homeostasis by regulating the storage of calcium ions. We explored the effects of EspF and the EspF–ANXA6 fusion protein on the uptake and release of calcium using the RHOD-2/AM fluorescent dye by laser confocal microscopy. RHOD-2/AM is a membrane-permeable calcium ion probe that enters cells and is hydrolyzed by intracellular esterases to release Rhod-2, which exhibits red fluorescence. Considering the potential impact of transfection on experimental outcomes, we designated the group transfected with the pEGFP plasmid as the negative control group. The image results indicate that the Ca^2+^ level in the negative control cells was lower than that in other groups. EspF-expressing cells presented a slight increase in the Ca^2+^ level compared with the negative control group. In addition, after transfection with the EspF–ANXA6 fusion plasmid for 48 h, the calcium fluorescence intensity in the cytoplasm of Caco-2 cells exhibited a further increase (Figure 4a,b). As above, EspF–ANXA6 interaction stimulates calcium release, which may aggravate the damage of ER homeostasis.

Since EspF–ANXA6 stimulated calcium release in Caco-2 cells, we next examined the role of EspF–ANXA6 in apoptosis. When cells were treated with the EspF–ANXA6 fusion plasmid, the average proportion of only Annexin V-positive cells (early apoptotic cells), and the proportion of both Annexin V-positive and PE-positive cells (late apoptotic cells), increased significantly, reaching approximately 1.85 ± 0.17% and 3.54 ± 0.21%, respectively (Figure 4c,d). These results demonstrate that the EspF–ANXA6 interaction induces Caco-2 cell apoptosis. Taken together, the obtained results indicate that EspF–ANXA6 interaction up-regulates the Ca^2+^ levels in the cytoplasm, leading to a calcium imbalance that aggravates the disruption of ER homeostasis and leads to cell apoptosis, which may eventually trigger the inflammatory response.

## 4. Discussion

Attaching and effacing pathogens like EHEC O157:H7 disrupt cell homeostasis by translocating effector molecules into the host cytoplasm, resulting in disease. Recent reviews of the pathogenesis of EHEC O157:H7 mostly focus on either Stx or adhesion [34,35]. However, effector proteins such as EspF are key regulators of EHEC O157:H7 [11]. A growing body of evidence suggests that a few metabolites released by *E. coli* and enteric pathogenic bacteria can cause ER dysfunction and induce proteins misfolding and unfolding to control the disease process [5,36]. Disturbance of the ER steady state is associated with inflammation and cell death. Stxs stimulate prolonged and severe ER stress in intestinal epithelial cells and induce cell death [37]. However, an additional study showed that an Stx-negative EPEC strain could still induce ER stress [38,39], which indicates that other regulators besides Stx can manipulate ER stress. It has been revealed that EspF could target the mitochondria, resulting in mitochondrial dysfunction, which, coupled with ER stress, may severely compromise epithelial integrity [40]. However, the precise mechanism and the role of ER stress in EspF-exposed cells have not been clearly verified. In a previous study, we identified EspF as a candidate regulator. Accumulating evidence has demonstrated that the interactions between host cells and pathogens are significant in the development of infectious diseases. Based on the strong positive mutual effect of EspF–ANXA6 and the function of ANXA6 in regulating the release of Ca^2+^, we proposed that the interaction between EspF and ANXA6 plays a role in ER stress.

First, we developed in vitro models employing Caco-2 cells infected with either wild-type EHEC or its Δ*espF* mutant to investigate the role of EspF in ER stress. BiP, an ER stress marker, plays a critical role in the ER protein quality control system, making adjustments to avoid the aggravation of protein misfolding under cellular stress [41]. Numerous bacteria, such as *Legionella pneumophila* and *Brucella*, can trigger ER stress as a critical strategy to maintain dissemination or long-term intracellular persistence by up-regulating BiP levels [38,42,43]. Nevertheless, the ER stress level is negatively affected by the presence of *Helicobacter pylori* [44]. According to previous studies, EHEC infection could obstruct protein transportation from the ER to the Golgi apparatus, blocking cell secretory pathways [45]. Our results reveal that EHEC O157:H7 infection facilitates ER stress to inhibit protein synthesis, which is consistent with these findings. However, infection with the *espF*-deletion O157:H7 strain increases BiP levels and stimulates the PERK pathway compared with cells undergoing EHEC O157:H7 infection. These findings support the idea that ER stress signals are reduced by EspF through the PERK pathway. Subsequently, we observed that EspF co-localizes with BiP in plasmid transfection models. Considering that BiP is a resident protein of the ER, this co-localization implies that EspF may be implicated in the regulation of ER stress. In our previous study [8], we observed a strong positive interaction between EspF and ANXA6 and found that their co-expression down-regulated the expression of tight junction proteins. In the current study, we further demonstrate that the interaction between EspF and ANXA6 alters the subcellular localization of EspF, causing it to migrate into the nucleus and weakening its co-localization with BiP. EspF–ANXA6 interaction may transfer stress signals from cytoplasmic receptors into the nucleus, thereby modulating CHOP levels and inducing ER-related apoptosis. We suspect that EspF–ANXA6 interaction further damages host cells from the cytoplasm to the nucleus.

The PERK pathway, one of three known UPR pathways, may serve as a therapeutic target to treat EHEC O157:H7-induced intestinal inflammation [20]. Due to the cytotoxicity of the transfection reagent, the high permeability, and the susceptibility to apoptosis of Caco-2 cells, the cells transfected with the pEGFP plasmid were regarded as the control group. We found that PERK pathway activity is significantly reduced in EspF- expressing and EspF–ANXA6-expressing cells. Our data indicate that EspF and EspF–ANXA6 block the PERK pathway, which enhances protein translation and promotes protein synthesis. It has been reported that *Simkania negevensis* infection could interfere with ER stress, which may result in delaying the clearance of bacteria and promoting the replication and proliferation of bacteria with an intracellular niche [46]. Considering the higher degree of evolution of bacteria compared to hosts, the inhibition of CHOP formation may slow down ER stress-related apoptosis to provide nutrition and a living environment for bacterial reproduction [29,46]. Interestingly, the observations in Caco-2 cells are quite different from our previous study in HT-29 cells [28] because of the differences in the expression levels of adhesion receptors and cell maturity [47,48]. Given the higher sensitivity of Caco-2 cells than HT-29 cells, we speculate that in the early stage of infection, EspF can down-regulate BiP levels to protect ER from imbalance, while in the late stage of infection, under constant ER stress, due to the compensation mechanism of the host cells, BiP levels are recovered. This hypothesis aligns with the findings of Lydia et al. [38], who reported that EPEC promotes the UPR during early infection, but UPR downstream target protein levels return to normal in the late stages of infection. In addition to the PERK pathway examined in this study, the other two branches of the UPR also play crucial roles in bacterial infections. The IRE1 pathway coordinates metabolic and immune responses, thereby regulating the differentiation of immune cells [49]. Meanwhile, the ATF6 pathway increases the expression of tumor necrosis factor (TNF) and inflammatory cytokines, thereby promoting the host inflammatory response [50].

ANXA6, as a member of the calcium- and membrane-binding annexins [8], could regulate calcium levels to activate cell signaling [51]. Based on our results, the interaction of EspF with ANXA6 could reduce the relatively high concentration of calcium within the ER lumen and then activate cell signaling responses. Some researchers believe that PERK mainly functions as a sensor of ER calcium levels [52]. Our results show that EspF–ANXA6 up-regulates cytoplasmic calcium levels by inhibiting the PERK pathway. With ER dysfunction, calcium homeostasis is disturbed, which can aggravate incorrect protein maturation and disturb numerous cellular processes, including metabolism, proliferation, differentiation, and transcription [53]. EspF–ANXA6 leads to a calcium ion transporter deficiency in the ER, eventually inducing apoptosis. Although the phosphorylation of eIF2α may activate CHOP production to induce apoptosis under many conditions [54], the question of whether the phosphorylation of eIF2α is intrinsically a pro- or anti-apoptotic signal remains controversial [55]. Taken together, we speculate that apart from the PERK pathway, other signaling pathways that control apoptosis next to EspF–ANXA6 exist.

Nevertheless, partial results cannot illuminate the precise mechanism clearly because of the complicated system of host cells. Also, due to the deficiencies in the co-expression system and the possible effects of the endogenous ANXA6 protein, the relationship between ANXA6 and ER stress still needs to be elucidated.

## 5. Conclusions

**Figure 5:**
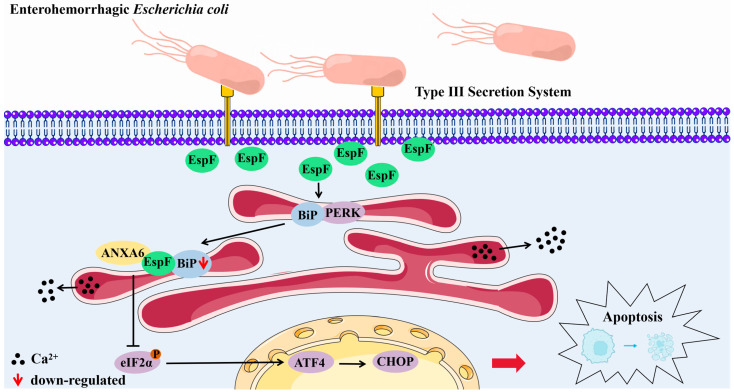
Model of the mechanism by which the EspF–ANXA6 interaction inhibits host ER stress. EspF, one of the effector proteins of EHEC O157:H7, communicates with ANXA6, reduces the expression level of BiP, inhibits the PERK pathway, increases calcium levels, inhibits ER stress, and induces apoptosis in Caco-2 cells.

In summary, we observed that (i) infection with the *espF*-deletion O157:H7 strain induces ER stress in Caco-2 cells, (ii) the decreased ER stress is dependent on the inhibition of the PERK pathway due to the interaction between EspF and ANXA6, and (iii) the EspF–ANXA6 interaction stimulates cellular calcium accumulation and induces apoptosis in Caco-2 cells. This demonstrates a novel role of the EspF–ANXA6 interaction, which inhibits ER stress through the PERK pathway to aggravate damage to the intestinal epithelial cells, disrupt host cell function, and promote bacterial survival (Figure 5).

## Data Availability

Data are contained within the article.

## Supplementary Materials

The following supporting information can be downloaded at https://www.mdpi.com/article/10.3390/pathogens14050440/s1. Supplementary Figure S1. Full-length gels of BiP protein and β-actin protein in Caco-2 cells infected with indicated bacteria strains for 6 h. Supplementary Figure S2. Full-length gels of eIF2α protein and p-eIF2α protein in Caco-2 cells infected with indicated bacteria strains for 6 h. Supplementary Figure S3. Full-length gels of ATF4 protein and β-actin protein in Caco-2 cells infected with indicated bacteria strains for 6 h. Supplementary Figure S4. Full-length gels of CHOP protein and β-actin protein in Caco-2 cells infected with indicated bacteria strains for 6 h. Supplementary Figure S5. Full-length gels of BiP protein and β-actin protein in HeLa cells transfected with indicated plasmids for 48 h. Supplementary Figure S6. Full-length gels of ANXA6 protein and β-actin protein in HeLa cells transfected with indicated plasmids for 48 h. Supplementary Figure S7. Full-length gels of CHOP protein and β-actin protein in HeLa cells transfected with indicated plasmids for 48 h. Supplementary Figure S8. Full-length gels of BiP protein β-actin protein in Caco-2 cells transfected with indicated plasmids for 48 h. Supplementary Figure S9. Full-length gels of ANXA6 protein and β-actin protein in Caco-2 cells transfected with indicated plasmids for 48 h. Supplementary Figure S10. Full-length gels of CHOP protein and β-actin protein in Caco-2 cells transfected with indicated plasmids for 48 h. Supplementary Figure S11. Full-length gels of eIF2α protein and p-eIF2α protein in Caco-2 cells transfected with indicated plasmids for 48 h. Supplementary Figure S12. Full-length gels of ATF4 protein and β-actin protein in Caco-2 cells transfected with indicated plasmids for 48 h.

## Abbreviations

The following abbreviations are used in this manuscript:

EHEC	Enterohemorrhagic *Escherichia coli*
*E. coli*	*Escherichia coli*
ANXA6	Annexin A6
ER	Endoplasmic reticulum
PERK	PKR-like ER kinase
Ca^2+^	Calcium
A/E	Attachment and effacement
Stxs	Shiga toxins
ESPs	*E. coli*-secreted proteins
LEE	Locus of Enterocyte Effacement
UPR	Unfolded Protein Response
Δ*espF*	*espF*-deletion strain
