# Supplementary material for: Enterohemorrhagic Escherichia coli O157:H7 Infection Inhibits Host Endoplasmic Reticulum Stress in Intestinal Epithelial Cells via the PERK Pathway
Source: Pathogens. 2025 Apr 30;14(5):440. doi: 10.3390/pathogens14050440 (PMC12114629; doi:10.3390/pathogens14050440)
Supplement: Supplementary file 1 [file pathogens-14-00440-s001.zip › Supplementary figure1-12.pdf]

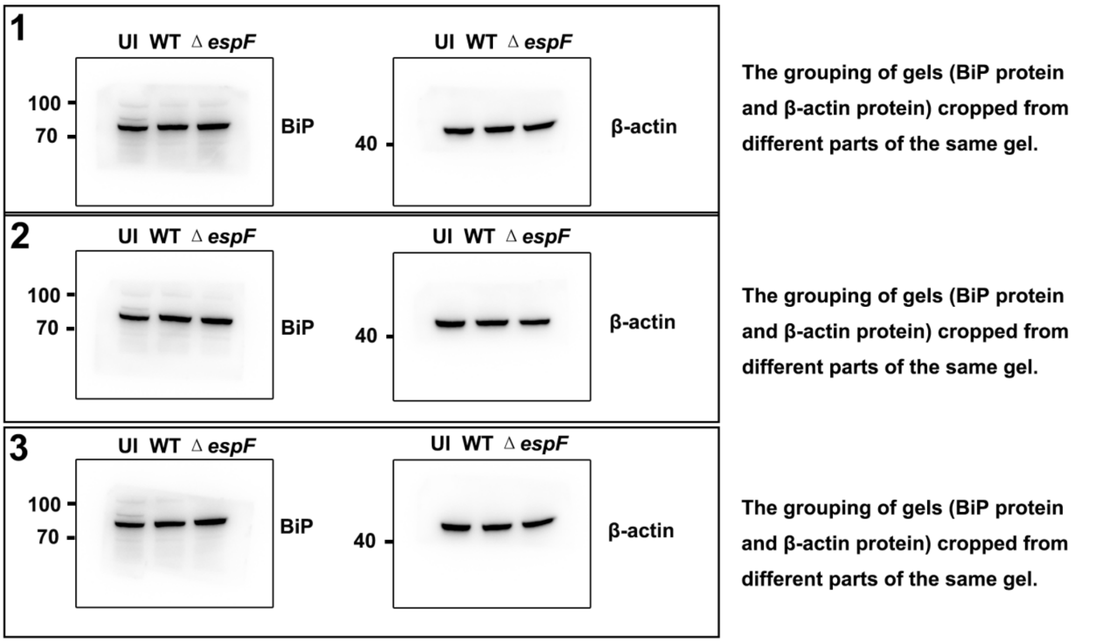

Supplementary figure S1. Full-length gels of BiP protein and β-actin protein in Caco-2 cells infected with indicated bacteria strains for 6 hours. UI, uninfected Caco-2 cells; WT, Caco-2 cells infected with EHEC O157:H7 strains EDL933 and Δ*espF*, Caco-2 cells infected with *espF*-deletion O157:H7 strains. The following three figures are the repeated experiments results. The grouping of gels (BiP protein and β-actin protein) cropped from different parts of the same gel.

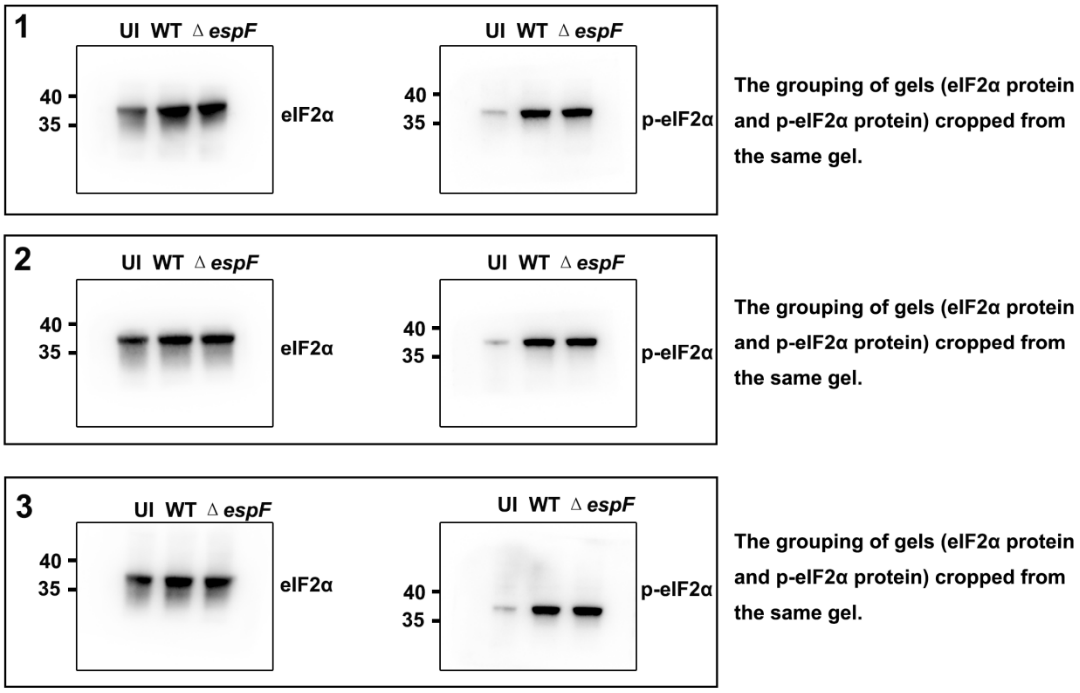

Supplementary figure S2. Full-length gels of eIF2α protein and p-eIF2α protein in Caco-2 cells infected with indicated bacteria strains for 6 hours. UI, uninfected Caco-2 cells; WT, Caco-2 cells infected with EHEC O157:H7 strains EDL933 and Δ*espF*, Caco-2 cells infected with *espF*-deletion O157:H7 strains. The following three figures are the repeated experiments results.The grouping of gels (eIF2α protein and p-eIF2α protein) cropped from the same gel.

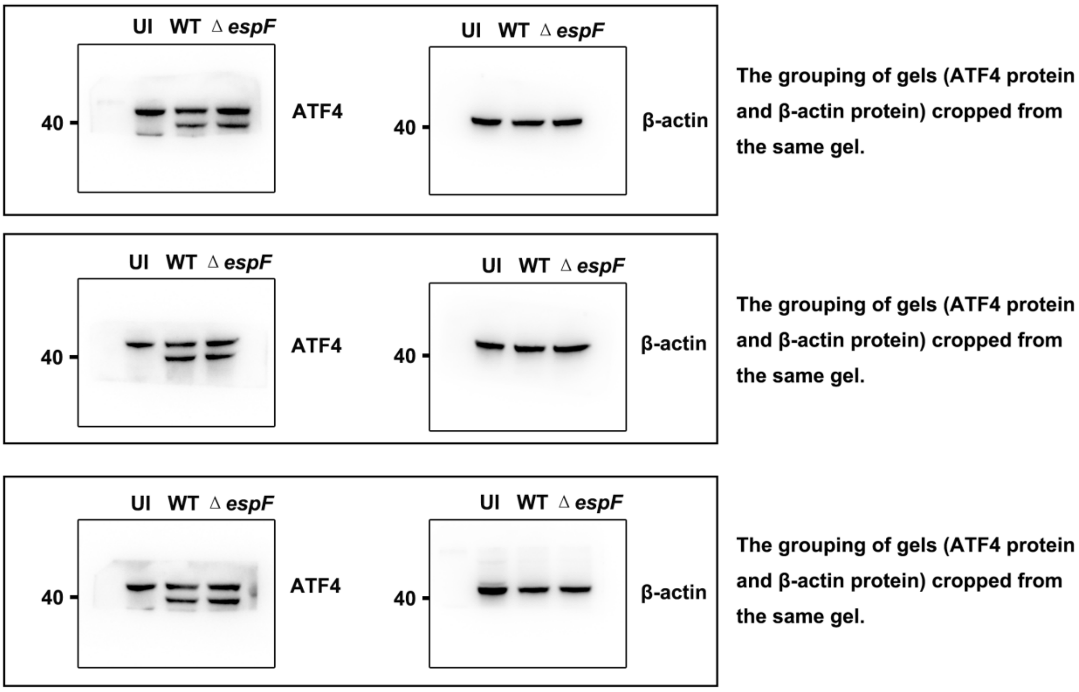

Supplementary figure S3. Full-length gels of ATF4 protein and β-actin protein in Caco-2 cells infected with indicated bacteria strains for 6 hours. UI, uninfected Caco-2 cells; WT, Caco-2 cells infected with EHEC O157:H7 strains EDL933 and Δ*espF*, Caco-2 cells infected with *espF*-deletion O157:H7 strains. The following three figures are the repeated experiments results.The grouping of gels (ATF4 protein and β-actin protein) cropped from the same gel.

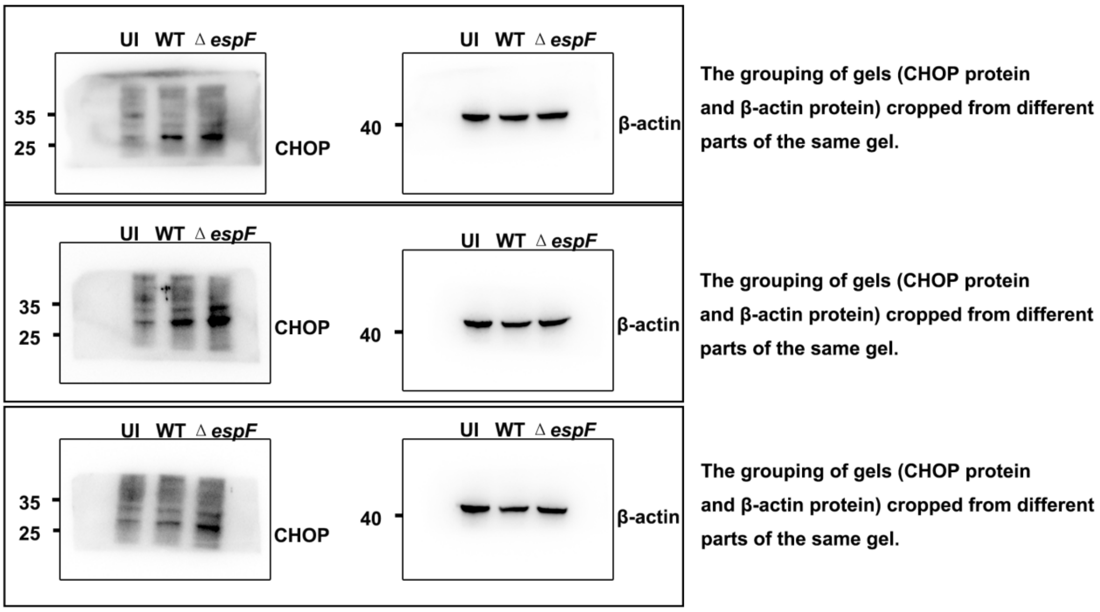

Supplementary figure S4. Full-length gels of CHOP protein and β-actin protein in Caco-2 cells infected with indicated bacteria strains for 6 hours. UI, uninfected Caco-2 cells; WT, Caco-2 cells infected with EHEC O157:H7 strains EDL933 and Δ*espF*, Caco-2 cells infected with *espF*-deletion O157:H7 strains. The following three figures are the repeated experiments results. The grouping of gels (CHOP protein and β-actin protein) cropped from different parts of the same gel.

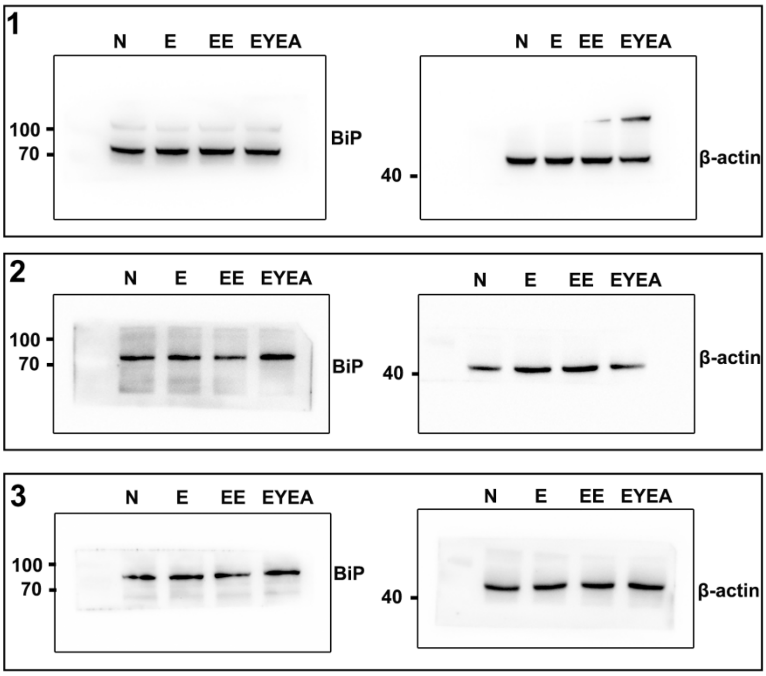

The grouping of gels (BiP protein and β-actin protein) cropped from different parts of the same gel.

The grouping of gels (BiP protein and β-actin protein) cropped from different parts of the same gel.

The grouping of gels (BiP protein and β-actin protein) cropped from different parts of the same gel.

Supplementary figure S5. Full-length gels of BiP protein and β-actin protein in HeLa cells transfected with indicated plasmids for 48 hours. (From left to right, there were group N, group E, group EE, and Group EYEA.) N, untransfected cells; E, cells transfected with pEGFP; EE, cells transfected with pEGFP-EspF; and EYEA, cells transfected with pEYFP-EspF-T2A-ANXA6. The following three figures are the repeated experiments results.The grouping of gels (BiP protein and β-actin protein) cropped from different parts of the same gel.

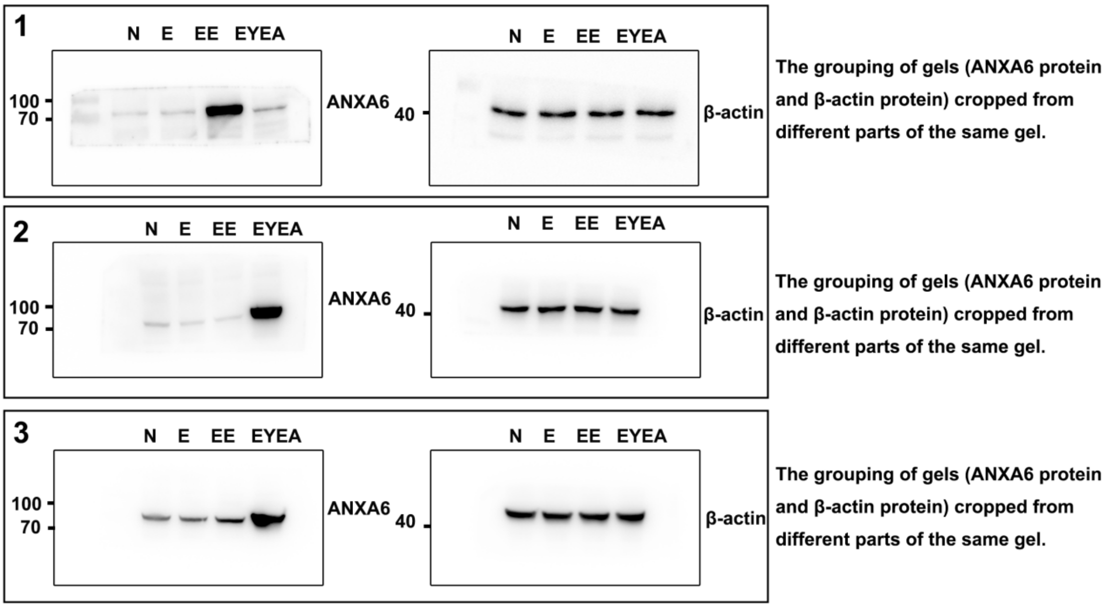

Supplementary figure S6. Full-length gels of ANXA6 protein and β-actin protein in HeLa cells transfected with indicated plasmids for 48 hours. N, untransfected cells; E, cells transfected with pEGFP; EE, cells transfected with pEGFP-EspF; and EYEA, cells transfected with pEYFP-EspF-T2A-ANXA6. The following three figures are the repeated experiments results. The grouping of gels (ANXA6 protein and β-actin protein) cropped from different parts of the same gel.

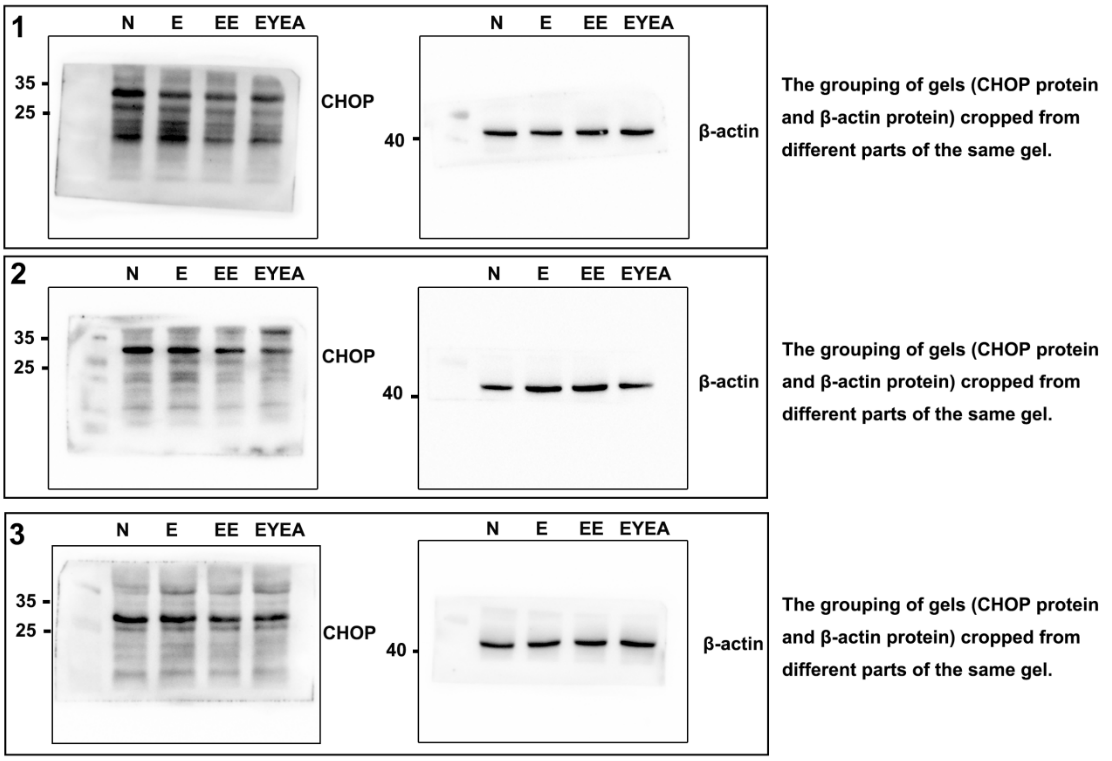

Supplementary figure S7. Full-length gels of CHOP protein and β-actin protein in HeLa cells transfected with indicated plasmids for 48 hours. (From left to right, there were group N, group E, group EE, and Group EYEA.) N, untransfected cells; E, cells transfected with pEGFP; EE, cells transfected with pEGFP-EspF; and EYEA, cells transfected with pEYFP-EspF-T2A-ANXA6. The following three figures are the repeated experiments results. The grouping of gels (CHOP protein and β-actin protein) cropped from different parts of the same gel.

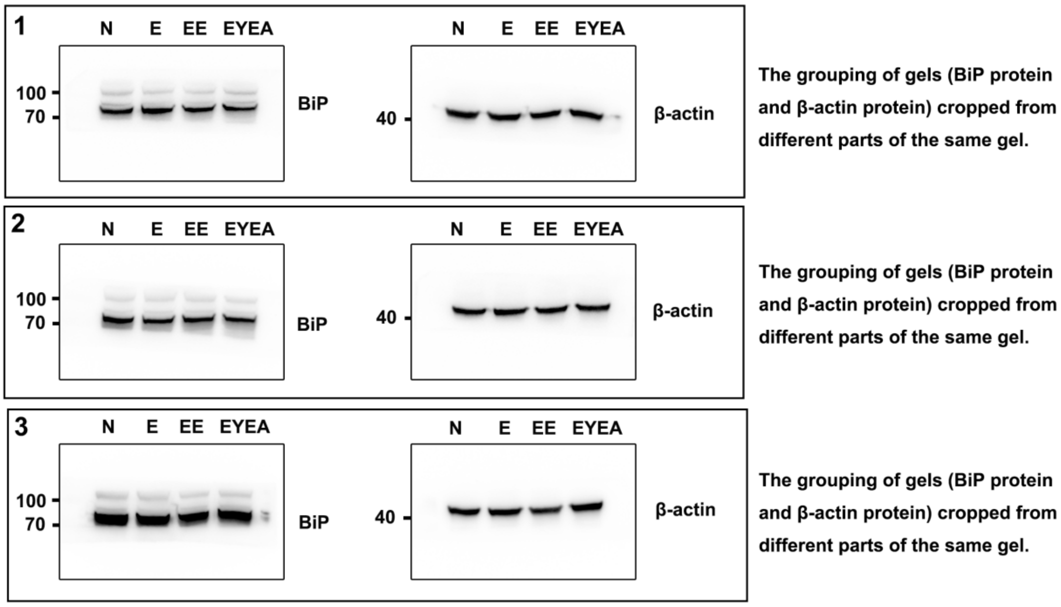

Supplementary figure S8. Full-length gels of BiP protein β-actin protein in Caco-2 cells transfected with indicated plasmids for 48 hours. N, untransfected cells; E, cells transfected with pEGFP; EE, cells transfected with pEGFP-EspF; and EYEA, cells transfected with pEYFP-EspF-T2A-ANXA6. The following three figures are the repeated experiments results. The grouping of gels (BiP protein and β-actin protein) cropped from different parts of the same gel.

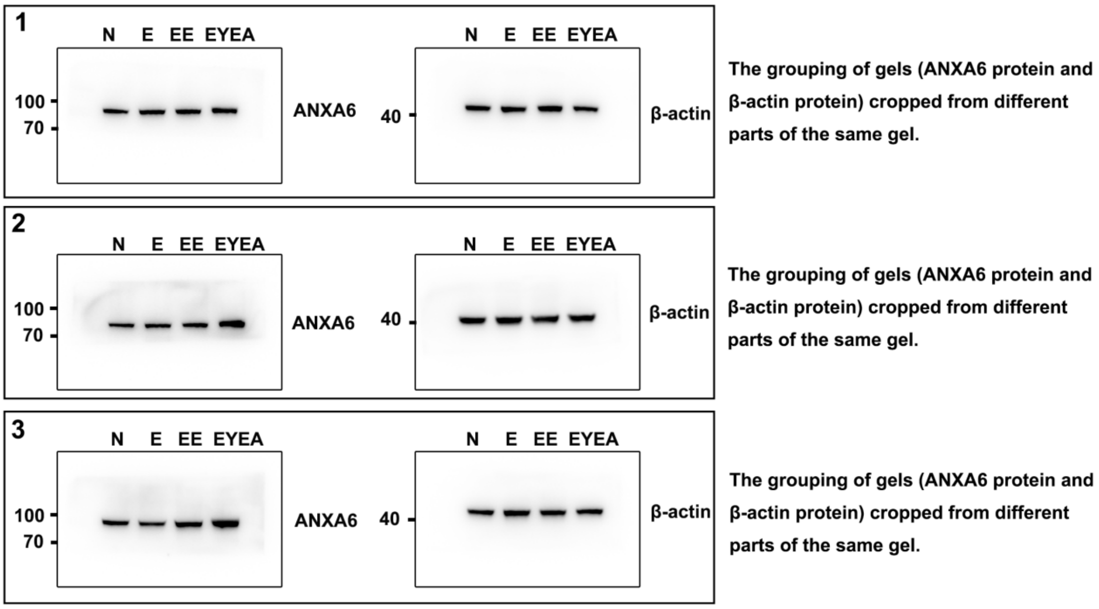

Supplementary figure S9. Full-length gels of ANXA6 protein and β-actin protein in Caco-2 cells transfected with indicated plasmids for 48 hours. N, untransfected cells; E, cells transfected with pEGFP; EE, cells transfected with pEGFP-EspF; and EYEA, cells transfected with pEYFP-EspF-T2A-ANXA6. The following three figures are the repeated experiments results. The grouping of gels (ANXA6 protein and β-actin protein) cropped from different parts of the same gel.

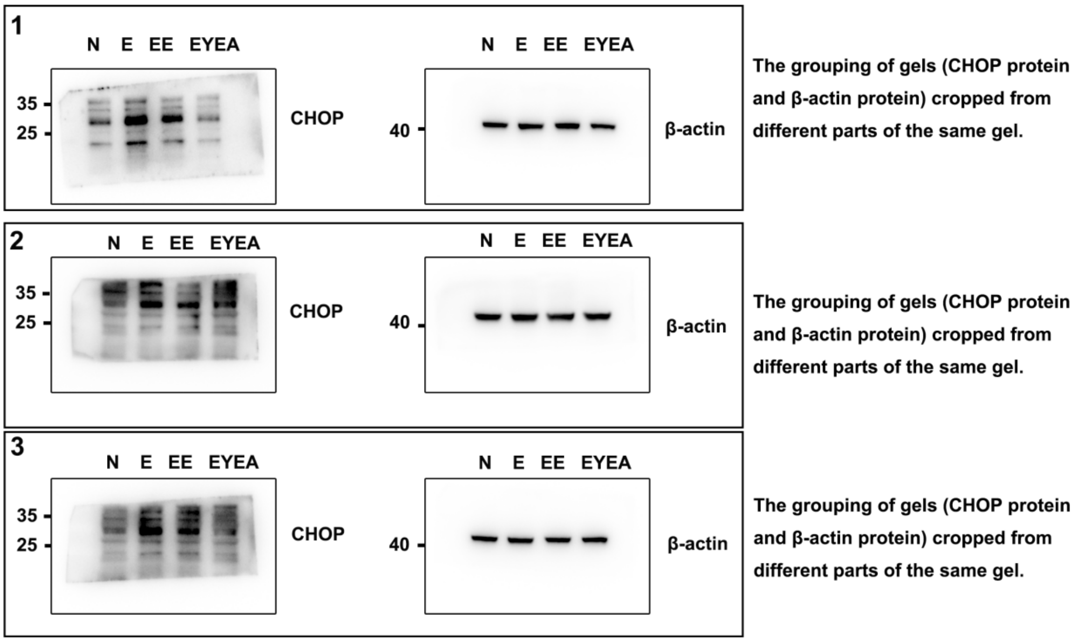

Supplementary figure S10. Full-length gels of CHOP protein and β-actin protein in Caco-2 cells transfected with indicated plasmids for 48 hours. N, untransfected cells; E, cells transfected with pEGFP; EE, cells transfected with pEGFP-EspF; and EYEA, cells transfected with pEYFP-EspF-T2A-ANXA6. The following three figures are the repeated experiments results. The grouping of gels (CHOP protein and β-actin protein) cropped from different parts of the same gel.

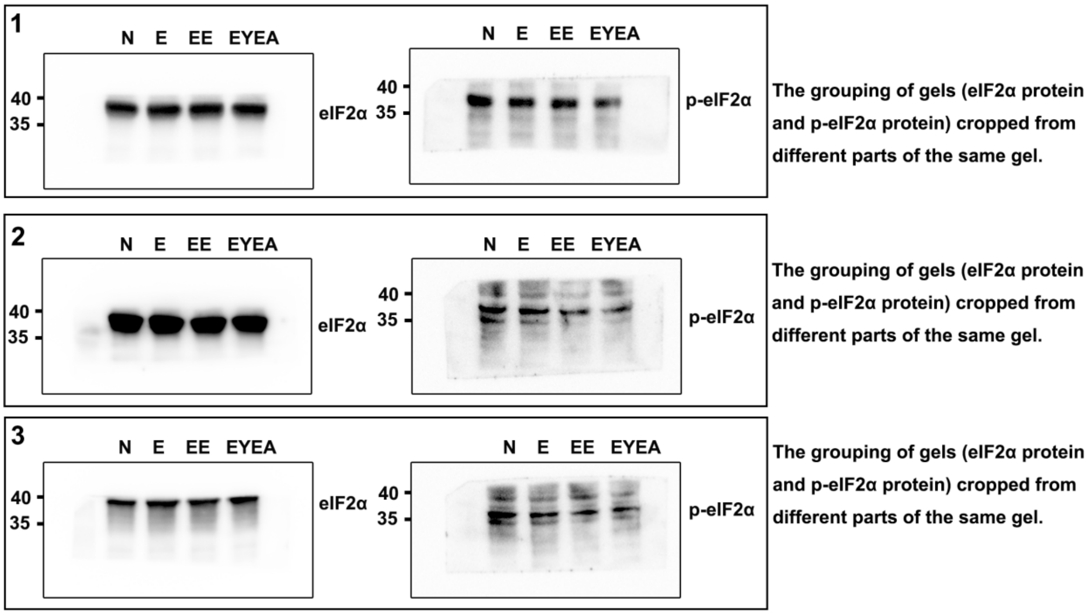

The grouping of gels (eIF2α protein and p-eIF2α protein) cropped from different parts of the same gel.

The grouping of gels (eIF2α protein and p-eIF2α protein) cropped from different parts of the same gel.

The grouping of gels (eIF2α protein and p-eIF2α protein) cropped from different parts of the same gel.

Supplementary figure S11. Full-length gels of eIF2α protein and p-eIF2α protein in Caco-2 cells transfected with indicated plasmids for 48 hours. (From left to right, there were group N, group E, group EE, and Group EYEA.) N, untransfected cells; E, cells transfected with pEGFP; EE, cells transfected with pEGFP-EspF; and EYEA, cells transfected with pEYFP-EspF-T2A-ANXA6. The following three figures are the repeated experiments results.The grouping of gels (eIF2α protein and p-eIF2α protein) cropped from the same gel.

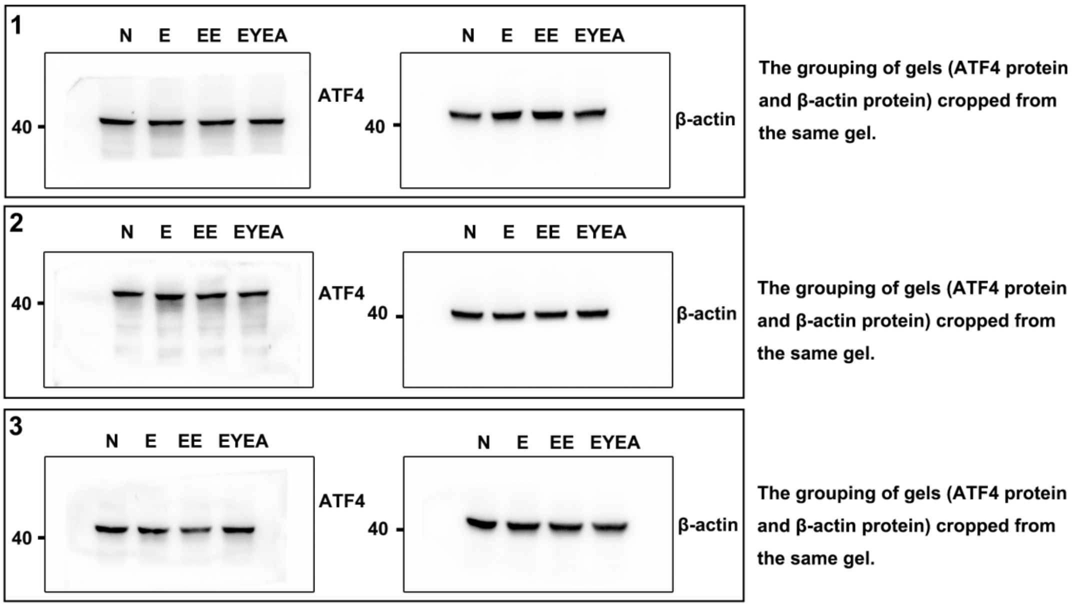

Supplementary figure S12. Full-length gels of ATF4 protein and β-actin protein in Caco-2 cells transfected with indicated plasmids for 48 hours. N, untransfected cells; E, cells transfected with pEGFP; EE, cells transfected with pEGFP-EspF; and EYEA, cells transfected with pEYFP-EspF-T2A-ANXA6. The following three figures are the repeated experiments results. The grouping of gels (ATF4 protein and β-actin protein) cropped from the same gel.
